# Salvage prostate therapies for local pelvic recurrence after primary radiation for prostate cancer

**DOI:** 10.1002/bco2.70259

**Published:** 2026-08-27

**Authors:** Jamie Michael, Kathryn Fink, Vivian Wan, Sai K. S. R. Kaushik, Clayton Neill, Yutai Li, Ridwan Alam, David J. VanderWeele, Shilajit Kundu, Ashley E. Ross

**Affiliations:** ^1^ Department of Urology, Feinberg School of Medicine Northwestern University Chicago Illinois USA; ^2^ Feinberg School of Medicine Northwestern University Chicago Illinois USA; ^3^ Department of Preventative Medicine, Feinberg School of Medicine Northwestern University Chicago Illinois USA; ^4^ Department of Hematology and Oncology, Feinberg School of Medicine Northwestern University Chicago Illinois USA

**Keywords:** cryoablation, oligo‐metastasis, prostate cancer, radiation, salvage

## Abstract

**Objectives:**

The purpose of this study is to evaluate salvage treatment after primary radiation therapy (RT) for prostate cancer within our hospital system. Specifically, we aim to evaluate treatment patterns, oncologic outcomes and complications.

**Materials and methods:**

We retrospectively identified men with post‐RT recurrence treated with salvage radical prostatectomy (sRP), seminal vesiculectomy, cryoablation, high‐dose‐rate brachytherapy (HDR) or irreversible electroporation (IRE). Salvage failure was defined as biochemical recurrence (BCR; Phoenix criteria, nadir + 2 ng/mL for cryoablation, HDR and IRE or prostate‐specific antigen (PSA) > 0.2 ng/mL for sRP and seminal vesiculectomy) or need for subsequent therapy. Cox regression identified factors associated with time to failure.

**Results:**

Seventy‐one patients underwent salvage therapy after RT; 58 (81.7%) cryoablation, 7 (9.9%) sRP, 2 (2.8%) robotic seminal vesiculectomies, and 4 (5.6%) other modalities. Failure occurred in 30 (45%) patients at a median of 12.5 months (IQR 6, 23). Grade group (GG) 4–5 disease prior to RT was associated with time to failure (HR 2.18, 95% CI 0.96, 4.97, *p* = 0.063) on multivariable analysis; however, the difference was not significant. Treatment type was not significantly associated with failure‐free survival (*p* = 0.7). Four patients experienced Clavien–Dindo grade III or higher complications: three following sRP (two anastomosis dehiscence, clot retention) and one following cryoablation (stricture). Ten patients (14%) underwent local salvage for prostate recurrences in the setting of N1/M1 disease with concurrent systemic therapy, with no treatment escalation or complications at median 16 months follow‐up time.

**Conclusions:**

In this institutional cohort, salvage cryoablation demonstrated an acceptable short‐term failure‐free survival with fewer observed high‐grade complications than salvage prostatectomy. Salvage cryoablation also appeared feasible in select patients with low‐volume nodal or metastatic disease. These findings are hypothesis‐generating and require prospective validation.

## 1 INTRODUCTION

Up to half of patients who undergo primary radiation therapy (RT) for prostate cancer (CaP) experience biochemical recurrence (BCR) within 10 years of definitive therapy.^1^ Biopsy studies also show that 15%–20% of patients treated with primary RT have residual disease, suggesting true local failure.^2^ Recurrence or local failure after RT is associated with worse overall survival, CaP‐specific survival and distant metastasis‐free survival.^3^

In contrast to recurrence after radical prostatectomy (RP), for which salvage RT is typically recommended because of its efficacy and favorable side effect profile, managing recurrence after RT remains challenging.^4^, ^5^ This is reflected in the historically low utilisation of treatment for local pelvic recurrences following RT,^6^ despite evidence that salvage local treatment can reduce prostate cancer specific mortality.^7^ Recent evidence suggests that treating the primary prostate tumour can improve symptoms and survival even in patients with synchronous oligometastatic disease.^8^, ^9^ At the same time, the emergence of more sensitive imaging modalities has increased the detection of oligometastatic presentations and improved our ability to differentiate localised recurrence from metastatic disease.^10^ Therefore, the treatment of local pelvic recurrences after primary RT may have even broader implications.

To reduce the morbidity associated with salvage radical prostatectomy (sRP) while preserving its potential mortality benefit, percutaneous ablative procedures and partial‐gland removals (seminal vesiculectomies) have emerged as favourable alternatives for salvage local pelvic recurrences after primary RT.^4^, ^5^, ^11^ Prior studies have reported comparable relapse‐free survival but lower toxicity when using these less invasive modalities.^7^

Given the expanding range of salvage options and the need to clarify their relative efficacy and morbidity profiles, it is essential to better define real‐world practice patterns and outcomes. Therefore, we aimed to describe institutional treatment patterns and explore associations among salvage modality, short‐term oncologic outcomes and treatment‐related complications.

## 2 MATERIALS AND METHODS

### 2.1 Study design and patient cohort

We performed a retrospective review of patients enrolled in a prospective, IRB‐approved registry of urologic outcomes who experienced failure after primary RT and subsequently underwent treatment for local pelvic recurrences between December 2009 and September 2025 at our tertiary medical centre (Northwestern Memorial Hospital). All patients received primary treatment with external‐beam radiation therapy, brachytherapy or a combination of both. Salvage procedures included in this analysis were sRP, seminal vesiculectomy, cryoablation, high‐dose‐rate brachytherapy (HDR) and irreversible electroporation (IRE). Treatment selection was individualised based on a combination of clinical factors, including lesion location on biopsy and imaging, prostate volume, extent of disease, prior radiation modality, patient comorbidity and baseline functional status and joint decision‐making between patient and surgeon. As seminal vesicles can be technically challenging to treat safely with ablative therapies, salvage vesiculectomy or prostatectomy was prioritised in cases where disease was either present in the seminal vesicles only or in the prostate and seminal vesicles, respectively. Salvage prostatectomy was typically reserved for younger, healthier patients. Among the ablative modalities, IRE and HDR were prioritised in patients with peri‐urethral lesions. Local pelvic recurrence was established by a combination of biopsy confirmation and imaging prior to salvage therapy. Pre‐salvage imaging was performed at provider discretion and evolved over the study period, reflecting real‐world practice patterns and more recent approval of PET imaging. Of the 71 patients, 43 (61%) underwent magnetic resonance imaging (MRI) prior to salvage, 22 (31%) underwent prostate‐specific membrane antigen positron emission tomography (PSMA PET) and 13 (18%) underwent both.

Salvage cryoablation was performed as whole‐gland ablation in 47 patients (81%) and as a subtotal gland ablation in 11 patients (19%). All procedures utilised the CryoCare CS platform (Siemens Healthineers). All procedures were performed using a urethral warming catheter, and monitoring was performed by transrectal ultrasound. Two freeze–thaw cycles were performed with target temperature reaching at least −40°C or lower. The procedure was not modified for patients with signs of local regional/systemic disease. sRP was performed robotically in six patients and via open approach in one patient. Seminal vesiculectomy was performed robotically in both patients. Prior to seminal vesiculectomy, disease localisation was determined by PSMA PET and biopsy in both cases, with one patient having isolated seminal vesicle recurrence and one with disease extending from the prostate base. HDR brachytherapy was performed using a targeted/focal approach in all patients, and the prescription dose to the gross tumour volume was the highest deliverable dose up to 30 Gy in one fraction. IRE was performed using a targeted/focal approach and procedure as previously described.^12^

### 2.2 Exposures and outcomes

Our primary exposure was type of salvage treatment received. Each salvage modality was evaluated individually. To further characterise the experience with cryoablation at our institution, we conducted an exploratory comparison between cryoablation and a composite group of all other salvage therapies; this pooling was driven by sample size constraints rather than an assumption of equivalence across modalities. The primary outcome was salvage treatment failure. Salvage treatment failure was defined as biochemical recurrence (using Phoenix criteria [PSA nadir + 2 ng/mL] for cryoablation, HDR and IRE, or prostate‐specific antigen (PSA) > 0.2 ng/mL for salvage radical prostatectomy and seminal vesiculectomy) or need for additional unplanned treatment. We assessed both the incidence of failure and failure‐free survival. Our secondary outcome was the occurrence of any treatment‐related complications.

### 2.3 Covariate measures

Covariates included demographic, clinical and procedural variables extracted from the medical record. Demographic variables consisted of age and Charlson Comorbidity Index (CCI). Baseline clinical characteristics include PSA prior to primary therapy, grade group (GG) on biopsy before primary radiation therapy, prostate volume, GG on postradiation biopsy, time interval between primary and salvage therapy, PSA prior to salvage therapy, use of androgen deprivation therapy (ADT) during salvage treatment, presence of nodal or metastatic disease at the time of salvage therapy, and PSA nadir following salvage treatment.

### 2.4 Statistical analysis

The patient characteristics were compared across salvage treatment types with Kruskal–Wallis for continuous variables and Fisher exact tests for categorical variables. Cox proportional hazards regression was used to evaluate factors associated with time to failure. A multivariable Cox model was constructed based on variables identified a priori.^13^ Proportional hazards assumption was assessed using Schoenfeld residuals and was met for all covariates. Results are presented as hazard ratios (HR) with 95% confidence intervals. Kaplan–Meier survival curves were generated to visualise failure‐free survival across salvage treatment groups over time. Survival differences between the salvage treatment groups were compared using the log rank test. Statistical significance was defined as *p* < .05. All statistical analyses were performed using R v4.5.1.

## 3 RESULTS

We identified 71 patients who received salvage therapy for local pelvic recurrences after primary radiation therapy, with a median follow‐up of 19 months (IQR 10.5, 31). Median age at salvage therapy was 71 (IQR 65, 74), and median CCI was 7.0 (IQR 5, 9.5). There were no significant differences in prostate size, PSA or GG at initial diagnosis across salvage treatment groups (Table 1).

**TABLE 1:** Demographics, therapy characteristics and outcomes by salvage type.

Variable	Overall (*N* = 71)	Salvage cryoablation (*n* = 58)	Salvage RALP (*n* = 7)	Salvage seminal vesiculectomy (*n* = 2)	Other^a^ (*n* = 4)	*p*‐value^b^
Baseline characteristics						
Age, median (IQR)	71 (65, 74)	72 (65, 74)	70 (66, 72)	70.5 (67, 74)	67 (61, 70)	0.37
CCI, median (IQR)	7.0 (5, 9.5)	7 (5, 9)	6 (5.5, 12.5)	6 (5, 7)	7 (5, 10)	0.90
PSA pre‐RT, median (IQR)	7.1 (5.6, 9.5)	7.1 (5.5, 8.7)	10.3 (7.6, 12.0)	6.7 (6.4, 6.9)	5.6 (4.7, 8.2)	0.16
GG on pre‐RT biopsy^c^, *n* (%)						0.65
1–3	50 (77%)	41 (79%)	5 (71%)	1 (50%)	3 (75%)	
4–5	15 (23%)	11 (21%)	2 (29%)	1 (50%)	1 (25%)	
Prostate size median (IQR)	28.6 (21.3, 42.8)	28.2 (19.9, 45.5)	37 (30, 37.3)	25.6 (24.0, 27.1)	30.6 (17.3, 47.7)	0.81
Salvage therapy characteristics						
Time between RT and salvage median (IQR)	91 (64, 119)	91 (67, 119)	103 (61.5, 118.5)	69 (64, 74)	116 (75.5, 153)	0.74
GG on presalvage biopsy, *n* (%)						0.37
No grade	8 (11%)	6 (10%)	1 (14%)	0	1 (25%)	
1–3	31 (44%)	26 (45%)	2 (29%)	0	3 (75%)	
4–5	32 (45%)	26 (45%)	4 (57%)	2 (100%)	0	
PSA presalvage, median (IQR)	3.3 (2.4, 7.0)	3.3 (2.3, 7.0)	4.5 (2.1, 5.4)	2.4 (2.4, 2.5)	5.6 (5.0, 7.0)	0.30
Concurrent ADT, *n* (%)						0.16
No	51 (73%)	38 (67%)	7 (100%)	2 (100%)	4 (100%)	
Yes	19 (27%)	19 (33%)	0	0	0	
N1/M1, *n* (%)						0.72
No	61 (86%)	48 (83%)	7 (100%)	2 (100%)	4 (100%)	
Yes	10 (14%)	10 (17%)	0	0	0	
Outcomes						
PSA nadir postsalvage median (IQR)	0.25 (0.02, 0.9)	0.26 (0.02, 1.4)	0.2 (0, 0.2)	0.2 (0.2, 0.3)	0.7 (0.5, 4.2)	0.21
Failure, *n* (%)						**0.02**
No	36 (55%)	33 (62%)	3 (42.9%)	0	0	
Yes	30 (45%)	20 (38%)	4 (57.1%)	2 (100%)	4 (100%)	
Complications, *n* (%)						0.10
No	53 (79%)	44 (82%)	3 (42.9%)	2 (100%)	4 (100%)	
Yes	14 (21%)	10 (19%)	4 (57.1%)	0	0	
Follow‐up time (m) median (IQR)	19 (10.5, 31)	16 (10, 27)	42 (32, 57)	14 (6, 22)	71 (45.5, 86.5)	**<0.001**
Time to failure	12.5 (6, 23)	12.5 (6.5, 22.5)	6.5 (4.5, 9.5)	13.5 (6, 21)	30 (26, 43.5)	**0.02**

Abbreviations: ADT, androgen deprivation therapy; GG, grade group; IQR, interquartile range; PSA, prostate‐specific antigen; RALP, robotic‐assisted laparoscopic prostatectomy; RT, radiation therapy.

^a^ Irreversible electroporation^1^ and high‐dose rate brachytherapy^3^.

^b^ *p*‐values calculated using Fisher–Freeman–Halton exact test for categorical variables and Kruskal–Wallis for continuous variables.

^c^ Preradiation grade group was unavailable in six patients and ADT status in one patient because of incomplete records. Failure status was indeterminate in five patients because of insufficient follow‐up. Complications data were missing in four patients.

Most patients underwent salvage cryoablation (82%), followed by sRP (10%), seminal vesiculectomy (3%), HDR (4%) and IRE (2%). Median time from primary radiation to salvage treatment was 91 months, with no significant differences between groups. All 71 patients underwent presalvage biopsy. Of these, 63 had an assigned GG: 2 (3%) had GG 1 disease, 11 (17%) had GG2, 18 (29%) had GG3, 18 (29%) had GG4 and 14 (22%) had GG5. In the remaining eight patients, a GG could not be assigned because of postradiation treatment changes.

### 3.1 Oncologic outcomes

Treatment failure occurred in 30 patients at a median of 12.5 months after salvage treatment. Among the 30 patients whose treatment failed, 24 underwent additional therapy (13 doublet therapy, 5 ADT monotherapy, 5 repeat cryoablation and 1 chemotherapy).

**FIGURE 1:**
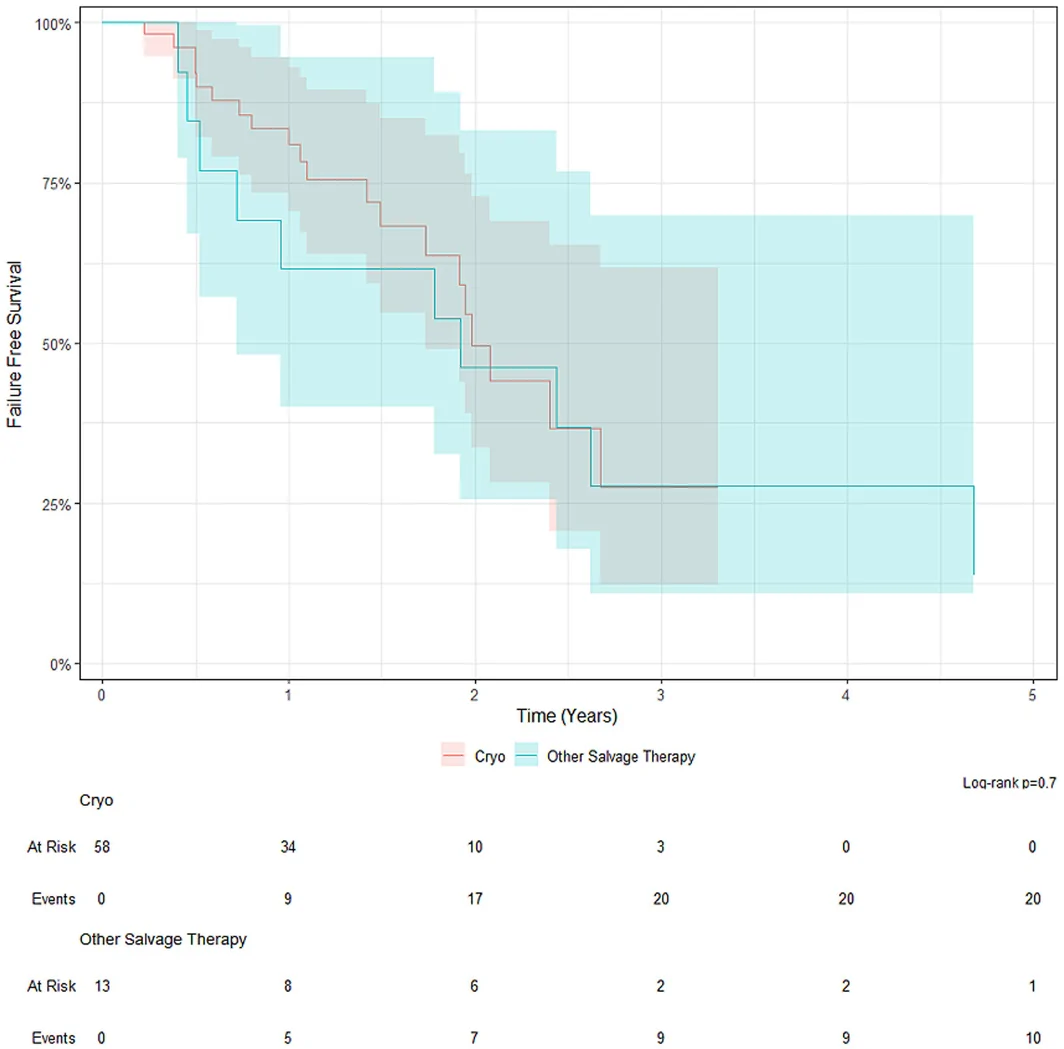
Failure‐free survival by type of salvage therapy.

One‐year failure‐free survival was 81% (95% CI 70%–93%) for cryoablation and 62% (95% CI 40%–95%) for other salvage modalities combined. At 2 years, failure‐free survival was 50% (95% CI 34%–73%) and 46% (95% CI 26%–83%), respectively. Failure‐free survival did not differ significantly between groups (log‐rank *p* = 0.7) (Figure 1). Salvage treatment type was not associated with failure‐free survival on either univariable or multivariable Cox regression (Table S1). On multivariable analysis, GG prior to RT was associated with worse failure‐free survival (HR 2.18, 95% CI 0.96%–4.97%); however, this was not statistically significant (Table 2).

**TABLE 2:** Multivariable Cox regression model of predictors of failure‐free survival.

Characteristic	HR	*p*‐value
GG Pre‐RT		
*GG1–3*	—	
*GG4–5*	2.18 (0.96, 4.97)	0.063
PSA presalvage	1.03 (0.96, 1.11)	0.4
Type of salvage		
*Cryo*	—	
*Other salvage therapy* ^a^	1.18 (0.50, 2.76)	0.7

Abbreviations: HDR, high‐dose‐rate brachytherapy; IRE, irreversible electroporation; RT, radiation therapy; sRP, salvage radical prostatectomy.

^a^ sRP, seminal vesiculectomy, HDR, IRE.

### 3.2 Salvage local therapy for metastatic or nodal disease

Ten patients underwent salvage cryoablation for local recurrence in the setting of nodal or metastatic disease and received concurrent systemic therapy. At a median follow‐up of 16 months, four patients were able to discontinue systemic therapy, two patients were able to de‐escalate from doublet therapy to ADT alone and four patients remained on systemic therapy. There were no cases of therapy escalation and no major complications.

### 3.3 Adverse events

We identified 14 treatment‐related complications: 4 (57%) in the sRP group and 10 (19%) in the cryoablation group. Four patients experienced complications that were Clavien–Dindo grade III or higher. In the sRP group, high‐grade complications included vesicourethral anastomotic (VUAS) dehiscence with clot retention requiring clot evacuation, catheter balloon rupture requiring foreign body removal and hematemesis requiring endoscopy. There were three total occurrences of anastomotic leak in the sRP cohort. In the cryoablation group, one patient developed a stricture requiring operative intervention (Table 3). No complications occurred among patients who underwent salvage cryoablation in the setting of metastatic or nodal disease.

**TABLE 3:** Complications by treatment group and Clavien–Dindo grade^a^

Grade	Complication	Management
Cryoablation (*n* = 10 complications in 10 patients)^b^		
I	Emergency room visit for catheter issue (*n* = 4)	Pain control
	Rash, lower extremity burning sensation	Conservative management
II	UTI (*n* = 3)	Antibiotics
IIIa	Stricture	Cystoscopy, dilation
Salvage prostatectomy (*n* = 7 complications in four patients)^c^		
I	Bladder leak, AKI	Prolonged foley duration
IIIb	VUAS dehiscence (*n* = 2), clot retention, catheter balloon rupture, hematemesis	Cystoscopy, clot evacuation, foreign body removal, endoscopy

^a^ Table reports complications; individual patients may have experienced more than one complication.

^b^ One complication per patient; 19% complication rate (10/54 evaluable).

^c^ One or more complications per patient; 57.1% complication rate (4/7).

## 4 DISCUSSION

Men treated with RT for prostate cancer tend to be older with more comorbidities than those initially treated with surgery, rendering the toxicity of salvage therapies particularly consequential.^14^ Salvage prostatectomy is historically associated with high morbidity, although modern robotic approaches have demonstrated improved complication rates.^15^, ^16^ Consequently, up to 90% of patients with recurrence after RT receive only ADT, foregoing potentially curative local salvage therapy.^6^ Percutaneous ablative procedures and partial gland removals have emerged as alternatives aimed at reducing morbidity while preserving the oncologic benefit of treating local pelvic recurrences in both localised and metastatic settings.^7^

In this study, we identified 71 men treated for local pelvic recurrences after primary RT. A majority of men underwent cryoablation (82%) followed by sRP, HDR, seminal vesiculectomy and IRE. Consistent with AUA guidelines,^5^ all patients underwent biopsy prior to salvage therapy to confirm recurrence, given the significant morbidity associated with these interventions.^17^, ^18^ High‐grade disease (GG4–5) was observed in 45% of post‐treatment biopsies. Historical practices discouraged grading postradiation biopsies; therefore, AUA and EAU guidelines rely on pre‐RT GG for risk stratification.^4^, ^5^ At our institution, GG was assigned in 63 of 71 cases, allowing us to explore its prognostic relevance. Baseline pre‐RT GG showed an association approaching significance with treatment failure, whereas presalvage GG was not predictive, suggesting that original tumour biology may remain more informative.

Overall, 30 patients (45%) experienced treatment failure at a median of 12.5 months postsalvage. Failure‐free survival did not differ significantly between salvage modalities on either univariable or multivariable analysis. These findings are consistent with prior studies reporting similar relapse‐free survival across modalities, although direct comparisons remain limited by study design and failure definitions.^7^, ^19^, ^20^, ^21^, ^22^ Differences in failure definitions, such as Phoenix criterion versus PSA > 0.5 ng/mL, complicate direct comparisons, highlighting a broader need for standardised outcome measures in salvage therapy research.

Most previous studies of various salvage modalities exclude patients with metastatic disease. In this cohort, 10 patients with low‐volume nodal or metastatic disease underwent local cryoablation combined with systemic therapy. At a median follow‐up of 16 months, no patients required escalation of therapy because of progression or treatment‐related complications, and four were off systemic therapy at last follow‐up. These observations are hypothesis generating and should be interpreted cautiously given the small number of patients and limited follow‐up. The diagnosis of oligometastatic disease has increased with the increased utilisation of higher sensitivity PET imaging,^23^ introducing complexity to the treatment of prostate cancer recurrences, as traditional dogma states that in the setting of metastatic disease there is no role for treatment of the prostate.^24^ Recent evidence from randomised clinical data demonstrates a survival benefit with radiation to the primary tumour in the setting of oligometastatic disease for patients with no prior primary treatment.^8^ Furthermore, multiple randomised trials have demonstrated that focal treatment of metastasis in the setting of recurrent oligometastatic disease can lead to extended time off systemic treatment.^25^, ^26^ These trials do not directly validate salvage cryoablation of an irradiated primary in recurrent N1/M1 disease and should not be extrapolated to support such an approach. Current guidelines endorse local salvage therapy only in the absence of metastatic disease,^4^, ^5^ and further prospective investigation is needed before any conclusions regarding its role can be drawn.^24^

The patient selection remains critical given the historically high toxicity of salvage therapies. Consistent with prior reports,^7^, ^27^ sRP was associated with the highest rate of serious complications, including vesicourethral anastomotic leaks and clot retention. In this cohort, less invasive salvage options, particularly cryoablation, were associated with fewer high‐grade complications, consistent with prior reports. However, the nonrandomised design and small comparator groups preclude definitive conclusions about comparative efficacy. Further, we acknowledge that functional outcomes, including urinary continence, erectile function and bowel function, were not systematically captured in this cohort because of inconsistent reporting across providers and the retrospective study design. This represents a meaningful limitation, as functional morbidity is central to salvage treatment decision‐making, particularly in older patients with baseline comorbidities. Future prospective studies should incorporate validated patient‐reported outcomes measures.

This study has several limitations. First, the sample size is modest and follow up was limited, affecting the generalisability and statistical power. The retrospective design introduces inherent selection and reporting biases. For example, treatment selection was nonrandomised and influenced by multiple clinical and provider‐level factors, presenting a potential source of selection bias that limits interpretation of between‐group comparisons. Follow‐up duration was substantially imbalanced across treatment groups, which limits direct cross‐group comparison of failure‐free survival estimates and precludes meaningful assessment of late toxicity in the cryoablation cohort. This imbalance reflects the predominance of cryoablation in more recent years of the study period and is an important caveat when interpreting between‐group comparisons. Nonetheless, this study provides novel insights into real‐world outcomes of salvage cryoablation, including in the setting of oligometastatic disease, and underscores the need for prospective studies with longer follow‐up.

## 5 CONCLUSIONS

In this single‐centre cohort study, salvage cryoablation was the most commonly used modality for local pelvic recurrence after primary RT and demonstrated acceptable short‐term failure‐free survival with fewer observed high‐grade complications than salvage prostatectomy. Because treatment assignment was nonrandomised, comparator groups were small and follow‐up was unequal across modalities, these findings should be considered hypothesis‐generating. Salvage cryoablation also appeared feasible in select patients with low‐volume metastatic or nodal disease, although this likewise requires prospective validation. Future prospective studies with larger cohorts and extended follow‐up are warranted to validate these results and clarify the role of local salvage therapy in the oligometastatic setting.

## AUTHOR CONTRIBUTIONS

*Conceptualisation*: A.E. Ross, *Methodology*: A.E. Ross. *Software*: C. Neill and Y. Li, *Validation*: J. Michael, K. Fink and A.E. Ross. *Formal analysis*: S.K.S.R. Kaushik. *Investigation*: J. Michael, Fink, K. and A.E. Ross. *Resources*: A.E. Ross. *Data curation*: J. Michael, K. Fink, C. Neill and Y. Li. *Writing* – *Original draft*: Michael, J. *Writing* – *Review and editing*: J. Michael, K. Fink, V. Wan, S.K.S.R. Kaushik, C. Neill, Y. Li, R. Alam, D.J. VanderWeele, S Kundu and A.E. Ross. *Visualisation*: S.K.S.R. Kaushik. *Supervision*: A.E. Ross. *Project administration*: J. Michael. *Funding acquisition*: A.E. Ross.

## CONFLICT OF INTEREST STATEMENT

The authors have no conflicts of interest to declare that are relevant to the content of this article.

## Supporting information


**Table S1.** Univariate regression analysis of factors associated with failure‐free survival.

## Data Availability

The deidentified database utilised for study analyses is available from the contributing author upon request.
