# Supplementary material for: Salvage prostate therapies for local pelvic recurrence after primary radiation for prostate cancer
Source: BJUI Compass. 2026 Aug 27;7(9):e70259. doi: 10.1002/bco2.70259 (PMC13522389; doi:10.1002/bco2.70259)
Supplement: Supplementary file 1 [file BCO2-7-e70259-s001.docx]

Supplemental Table 1 – Univariate regression analysis of factors associated with failure-free survival

| **Characteristic** | **HR (95% CI)** | **p-value** |
| --- | --- | --- |
| **Age** | 0.99 (0.94, 1.05) | 0.8 |
| **CCI** | 0.96 (0.85, 1.09) | 0.5 |
| **PSA pre-salvage** | 1.03 (0.96, 1.11) | 0.4 |
| **Salvage type** |  | 0.7 |
| *Cryoablation* | — |  |
| *RP* | 0.95 (0.31, 2.93) | >0.9 |
| *Seminal vesiculectomy* | 3.49 (0.80, 15.3) | 0.10 |
| *IRE* | 0.96 (0.13, 7.25) | >0.9 |
| *HDR* | 0.94 (0.26, 3.41) | >0.9 |
| **Prostate volume** | 1.01 (1.0, 1.02) | 0.3 |
| **GG Pre-RT** |  | 0.071 |
| *GG1-3* | — |  |
| *GG4-5* | 2.21 (0.98-5.02) | 0.057 |
